# Determination of Organophosphorus Esters in Fall Protection Equipment by Accelerated Solvent Extraction and Solid-Phase Extraction Coupled with LC-MS/MS Detection

**DOI:** 10.1155/2021/8878247

**Published:** 2021-01-05

**Authors:** Haihong Li, Mingli Ye, Fangfang Wu, Xuyang Zhao, Lifeng Wang, Yili Wei, Shengyi Xie, Hairong Cui

**Affiliations:** ^1^Zhejiang Key Laboratory for Protection Technology of High-Rise Operation, Zhejiang Huadian Equipment Testing Institute Co., Ltd, Hangzhou, Zhejiang 310015, China; ^2^SGCC-Testing Technology Lab of Electrical Equipment Safety Performance, Hangzhou, Zhejiang 310015, China; ^3^School of Life Science, Wuchang University of Technology, Wuhan, Hubei 430223, China; ^4^College of Biological and Environmental Engineering, Zhejiang Shuren University, Hangzhou 310015, China

## Abstract

An analysis method was established to determine 14 organophosphorus ester (OPE) flame retardants in fall protection equipment by combining accelerated solvent extraction (ASE) and solid-phase extraction (SPE) with high-performance liquid chromatography-tandem mass spectrometry (HPLC-MS/MS). The ASE parameters were optimized as follows: static extraction with acetonitrile at 80°C for 5 min for two cycles. The combined extract was purified with the ENVI-18 cartridge before further analysis. A HILIC column was used to separate the OPEs using an acetonitrile/water mixture as the mobile phase with the detection by the electrospray ionization mass spectrometry, which was operated under the positive mode. Under optimized conditions, the limit of detection for the target OPEs ranged in 0.015–1.33 ng/g, with a spike recovery of 71.6%–114% and a relative standard deviation of 0.8%–11.2%. The developed method was used to analyze OPEs in fall protection equipment (safety helmets and ropes), where OPEs were all detectable. Safety ropes displayed a higher concentration of OPEs than ones in safety helmets, with the pollutants being mainly triphenyl phosphate, 2-ethylhexyl diphenyl phosphate (EHDPP), tri(2-ethylhexyl) phosphate, and tri-n-butyl phosphate in the range of 11.07 ng/g‒815.53 ng/g. The EHDPP was the dominant compound in safety helmets with the concentration from 26.84 to 95.29 ng/g, while the other OPEs in safety helmets were lower than 5.136 ng/g. The potential health and environmental risks of these fall protection equipment during their use and disposal call for further attention.

## 1. Introduction

After the use of polybrominated diphenyl ethers (PBDEs) is banned worldwide, organophosphorus esters (OPEs), as flame retardants with excellent properties and abundant resources, are now widely used in plastics, furniture, textiles, electronic equipment, building materials, automobiles, etc. [1]. Both the demand and the production of OPEs have increased significantly in recent years with the worldwide restriction of PBDEs and the gradual implementation of the mandatory national standard “Flame retardant products and components in public places-requirements and marking for combustion performance” (GB 2028-2006) in China. The global use of OPEs reached 341,000 t in 2007 [2] and increased to 680,000 t in 2015 [3]. The annual production of OPEs was estimated to be about 70,000 t in 2007 and be growing at a rate of 15% per annum in China [1]. Existing studies have demonstrated that some OPEs adversely affect organisms and humans. For example, chlorinated OPEs, including tri(2-chloroethyl) phosphate (TCEP), tri(1-chloro-2-propyl) phosphate (TCPP), and tri(1, 4443-dichloro-2-propyl)phosphate (TDCPP), are found to be potentially carcinogenic and can cause tumor growth in multiple organs such as liver, kidney, thyroid, and brain [1]. In addition, tri-n-butyl phosphate (TnBP), TCPP, TDCPP, and trimethylphenyl phosphate (TMPP) interfere with the thyroid hormone [4]. TDCPP inhibits DNA synthesis and has certain neurotoxicity [5]. Some OPEs, such as triphenyl phosphate (TPhP), TMPP, and TDCPP, have estrogen-inducing effects, whereas TCEP and tri(2-ethylhexyl) phosphate (TEHP) have significant antiestrogen effects [6]. Moreover, some aromatic OPEs have been shown to cause DNA damage as well as mitochondrial damage [7].

In recent years, OPEs have been detected by a number of studies in home furniture foam [8], plastic appliances [9], construction and decoration materials [10], and textile supplies [11]. As we know, fall protection equipment generally uses plastic or textile materials with high mechanical strength to ensure personal safety at height. Flame retardants are added in the manufacturing of fall protection equipment such that the equipment can prevent, delay, or terminate the spread of flames in case of external fire and protect the life and health of workers. For example, a qualified safety helmet must not burn for more than 5 s, and the shell of the helmet was not allowed to be burned through [12]. In particular, the equipment used by firefighters must be made from the highly retardant and reflective materials in order to protect their safety at work. OPEs tend to be released through volatilization, abrasion, or leaching from the safety products where they are added physically [1], consequently resulting in environmental pollution. Moreover, because many fall protection equipment (e.g., safety helmets and ropes) are in direct contact with human skin, the OPEs may be absorbed by humans and pose potential health risks. Additionally, inappropriate disposal of waste equipment may also cause environmental problems. Therefore, the accurate detection of OPEs in fall protection equipment is essential to the quality control of relevant products and to the amelioration of environmental risk.

Some works have studied the method for the detection of OPEs in different environmental matrices, such as water, air, dust, sediment, and biota. Commonly used detection methods include gas chromatography-tandem mass spectrometry (GC-MS) [13–15] and liquid chromatography-tandem mass spectrometry (LC-MS/MS) [16–18]. Compared with GC-MS, the LC-MS/MS method showed several advantages, such as low matrix effect resulting from good separation for complex samples, high sensitivity, and accuracy [19]. Therefore, it is widely used to investigate the environmental pollution of OPEs in various environmental media [20–22], atmospheric particulates [23], and foodstuff [24]. However, the extraction and detection of OPEs in fall protection equipment remain unreported. Accelerated solvent extraction (ASE), a kind of automated technique, can greatly shorten the extraction time and enhance the contact surface of the solvent sample via increasing the extraction temperature and pressure, as a result of improving the extraction efficiency. Therefore, since it was introduced in 1995, ASE has become a promising alternative technique of the traditional Soxhlet extraction method and has been widely used in the extraction of various organic pollutants (including OPEs) from environmental solid samples [25]. In this study, the materials (plastics and textiles) used in safety helmets and ropes are processed firstly by ASE and subsequently by conventional solid-phase extraction (SPE). In this way, an analytical method for OPEs in fall protection equipment was established with the combined use of ASE, SPE, and HPLC-MS/MS. This study thus supports the analysis of OPEs in fall protection equipment as well as the evaluation of associated health and environmental risks.

## 2. Materials and Methods

### 2.1. Chemicals and Standards

Table S1 lists the details of the 14 target analytes, including trimethyl phosphate (TMP), triethyl phosphate (TEP), tripropyl phosphate (TPrP), TnBP, tri-iso-butyl phosphate (TiBP), TEHP, TCEP, TCIPP, TDCPP, TPhP, TMPP, EHDPP, and cresyl diphenyl phosphate (CDPP). They were purchased from Dr. Ehrenstorfer GmbH (Germany). The isotope-labeled internal standards include d9-TMP, d15-TEP, and d21-TPrP, which were purchased from C/D/N Isotopes (Canada), and d27-TnBP and d15-TPhP, which were purchased from Cambridge Isotope Laboratories (USA). Another two internal standards d18-TCPP and d12-TCEP were purchased from Toronto Research Chemicals (Canada).

Methanol and acetonitrile (HPLC grade) were purchased from Merck (USA). Dichloromethane (HPLC grade) was obtained from Fisher Scientific (Pittsburgh, PA, USA). Ammonium acetate with high purity (>97%) was obtained from Alfa Aesar (Ward Hill, MA, USA). ENVI-18 cartridges (6 mL, 500 mg) were purchased from Supelco (USA). The Milli-Q ultrapure water preparation system was supplied by a Milli-Q Advantage A10 System (Millipore, USA).

### 2.2. Sample Pretreatment

The safety helmet and ropes were cut into small pieces (5 mm) and weighed (0.5 g and 1.0 g, respectively) and then placed into the extraction cell (10 mL) of the accelerated solvent extraction instrument (ASE 350, Dionex, Inc.). Then, 10 ng internal standards were added into the sample with thorough mixing. The bottom of the cell was installed with a filter membrane and then added a certain amount of processed diatomite (ground into powder and calcined at 450°C for 4 h) to fill the cell. The extraction was carried out with acetonitrile for 5 min at 80°C and 1500 psi for two cycles. After the volume of the extract was reduced to <0.5 mL by nitrogen blowing, ultrapure water (30 mL) was added, and the diluted extract was purified using the previously reported SPE method [18]. The cleanup process was performed as follows: the ENVI-18 cartridge was first conditioned successively with acetonitrile (5 mL) and ultrapure water (5 mL). The diluted extract was then loaded onto the conditioned cartridge. The cartridge was washed with ultrapure water (10 mL) after loading was completed and then was drained further under negative pressure for about 1 h. Afterwards, the targets were eluted with 1 : 3 v/v dichloromethane/acetonitrile (6 mL). The eluent was blown to near dryness under the gentle nitrogen and then prepared as a solution in water/acetonitrile (v : v; 50 : 50; 1 mL) and passed through a nylon filter membrane (0.22 *μ*m) before injection into HPLC-MS/MS.

### 2.3. Instrumental Analysis

The analysis of OPEs was conducted in the HPLC (UltiMate 3000, Thermo Fisher Scientific Co.) coupled with an electrospray ionization tandem mass spectrometry (ESI-MS/MS, API 3200, Applied Biosystems/MDS SCIEX, USA). An Analyst 1.6.2 workstation was used to process the data.

The chromatographic separation was carried out on an Acclaim Mixed-Mode HILIC-1 column (2.1 mm × 150 mm, 5 *μ*m; Thermo Fisher) using a binary mobile phase consisting of pure water (A) and acetonitrile (B) with a column temperature of 30°C at a flow rate of 0.25 mL/min using the following gradient elution procedure: 60% A at 0–5 min and then linear decreases from 60% A to 40% A over 5–8 min and to 0% A at 12 min. After phase A was returned to the original value of 60% over 12 to 15 min, the elution was maintained for 7 min in order to equilibrate the column.

The MS was operated under the positive ion and multiple reaction monitoring mode (MRM). The key parameters are as follows: curtain gas pressure, 0.14 MPa; collision gas pressure, 0.02 MPa; ion spray voltage, 5000 V; source temperature, 600°C; gas1, 0.34 MPa; and gas2, 0.28 MPa. Additional parameters are listed in Table S2.

## 3. Results and Discussion

### 3.1. Optimization of Sample Extraction

To optimize the extraction efficiency for OPEs in plastics and textiles, plastic and textile samples free of OPEs were blended with a mixture of standards (10 ng) and then subjected to ASE to investigate the key extraction parameters, including extraction solvent, temperature, and number of cycles. Generally speaking, among the abovementioned factors, the extraction solvent is the most important factor influencing the extraction efficiencies of OPEs. Therefore, it was firstly optimized under the fixed extraction temperature (80°C) and static cycles (3 cycles). After the solvent was determined, the extraction temperature was optimized under the fixed optimized solvent and static cycles (3 cycles). Finally, the static cycle times were revaluated under the optimized solvent and temperature.

#### 3.1.1. Selection of Extraction Solvent

**Figure 1:**
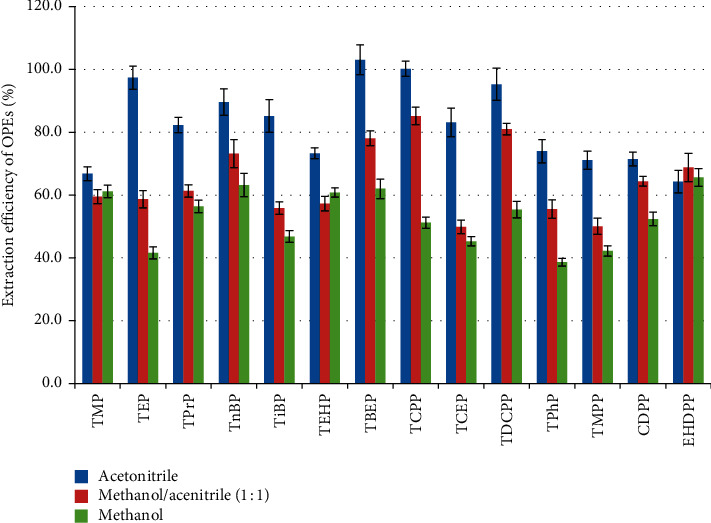
The effect of extraction solvent on the extraction efficiency of OPEs.

The extraction solvents, acetonitrile, methanol, and the mixture of methanol and acetonitrile (1 : 1, v/v), were optimized under the conditions at 80°C over 3 extraction static cycles.  Figure 1 shows the extraction efficiency of the three systems. As shown in Figure 1, the extraction efficiency of acetonitrile for the 14 kinds of targets is between 64.3% and 103.1%, which is significantly higher than that of methanol and the acetonitrile/methanol mixture using the statistical analysis (one-way ANOVA, *p* < 0.05). When methanol alone was used for extraction, the extraction efficiency of all substances was lower than 65%. Therefore, acetonitrile was chosen as the extraction solvent.

#### 3.1.2. Optimization of Extraction Temperature

**Figure 2:**
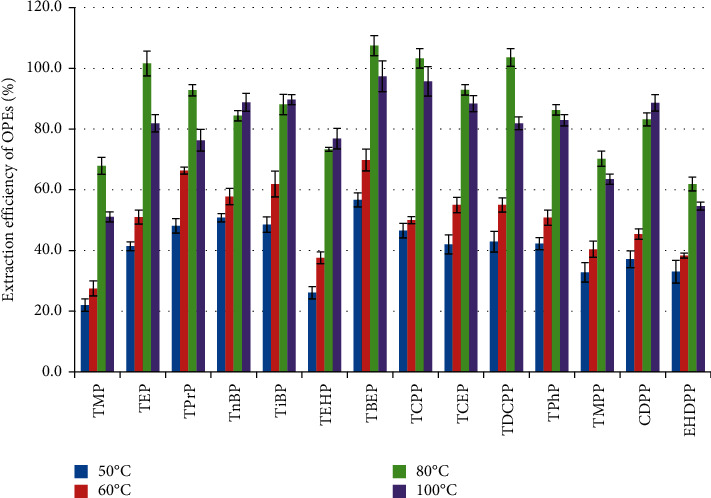
The effect of extraction temperature on the extraction efficiency of OPEs.

The extraction temperature was varied at 50, 60, 80, and 100°C while other extraction parameters were fixed (extraction solvent: acetonitrile; static cycles: 3).  Figure 2 shows that the extraction efficiency increased with rising extraction temperature. When the extraction temperature was greater than 60°C, the extraction efficiency of most OPEs improved significantly. However, the extraction efficiency of certain compounds declined when the temperature was increased to 100°C. It could be speculated that the viscosity of the solvent decreased with rising temperature, thereby enhancing the ability of the solvent to infiltrate the matrix and dissolve the target analyte. The additional thermal energy also helped to weaken the interaction between the target compound and the matrix, enhance the ability of the target analyte to diffuse from the matrix surface into the solvent, and thus improve the extraction efficiency. Nevertheless, impurity components also increased significantly when the temperature was increased to 100°C. The presence of impurities had an appreciable influence on the subsequent LC-MS/MS analysis, thereby reducing the extraction efficiency and reproducibility of the target analyte. Overall, when the extraction temperature was 80°C, the extraction efficiency of all OPEs except TMP, TEHP, and EHDPP ranged between 70.2% and 107.5%. In addition, the statistical analysis showed that there is a significant difference among the different temperatures (one-way ANOVA, *p* < 0.05). Therefore, the final extraction temperature was selected as 80°C.

#### 3.1.3. Optimization of Static Cycles

**Figure 3:**
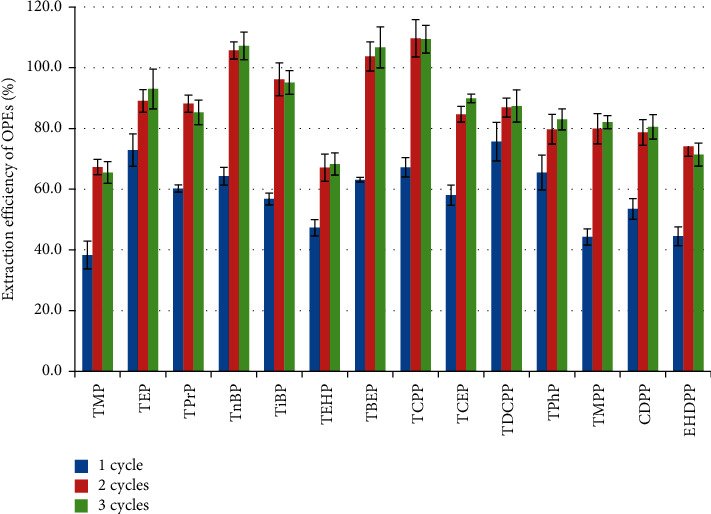
The effect of static extraction cycles on the extraction efficiency of OPEs.

The number of extraction cycles was varied at 1, 2, and 3 cycles while other extraction parameters were fixed.  Figure 3 shows the extraction efficiency of the 14 kinds of OPEs. After one extraction cycle, a large number of OPEs had only less than 60% extraction efficiency, whereas after two extraction cycles, all analytes other than TMP and TEHP had more than 75% extraction efficiency. Statistical analysis (one-way ANOVA) showed that the extraction efficiencies via two and three extraction cycles were significantly higher than ones based on only one cycle (*p* < 0.05), while there is no significant difference between two and three extraction cycles (*p* > 0.05), indicating that the third extraction cycle did not notably improve the extraction efficiency of OPEs. Therefore, two static cycles were considered as the optimal condition and used to extract OPEs from plastics and textiles.

### 3.2. Chromatographic Separation and Mass Spectrometry Conditions

The packing material of the Mixed-Mode HILIC-1 column has both hydrophobic alkyl chains and hydrophilic ethylene glycol end groups, thus allowing both hydrophobic retention and hydrophilic retention. The hydrophobic retention mechanism is dominant when the chromatography is operated in the reverse-phase mode. The hydrophilic ethylene glycol end groups enhance the retention of polar compounds and weaken the hydrophobic retention of nonpolar compounds, thereby effectively differentiating target compounds with varied physical and chemical properties. Consequently, multiple target analytes of diverse properties can be separated in relatively short time. The 14 kinds of OPEs in this study have large differences in various properties such as polarity, and their retention on the reversed-phase column differs strongly. Some strongly polar OPEs are poorly retained and thus not well separated from the matrix interference peaks, whereas some strongly hydrophobic components have low separation efficiency because of their longer retention time. Therefore, in this study, the Mixed-Mode HILIC-1 column was used for the HPLC-MS/MS analysis to account for the large polarity difference among the 14 kinds of target OPEs. Two kinds of combinations (acetonitrile with water and acetonitrile with 50 mmol/L ammonium acetate aqueous solution) were tested as the mobile phase with gradient elution. It was found that both systems could give sharp symmetrical peaks for all 14 OPEs, and the analytical sensitivity was high when acetonitrile/water was used. Therefore, the acetonitrile/water system was eventually chosen as the mobile phase for separation.

In addition, the MS-related parameters of 14 OPEs were optimized to obtain their optimal sensitivity and stability. The optimized mass spectrometry parameters, including qualitative and quantitative ion pairs, declustering voltage (DP), inlet voltage (EP), and collision voltage (CE), are listed in Table S2.

### 3.3. Standard Curve and Limit of Detection

The mixed standard solutions (0.1, 0.5, 1, 5, 10, 20, 50, 100, and 500 ng/mL) were measured under the optimized chromatographic and mass spectrometry conditions. The results showed that all 14 OPEs in the concentration range of 0.1–500 ng/mL showed a good linear response with a correlation coefficient of above 0.99. The limits of detection (LODs) were defined as the concentrations resulting in a signal-to-noise ratio of 3 (*S*/*N* = 3). The result showed that the LODs of the 14 OPEs ranged in 0.015–1.33 ng/g (Table 1).

### 3.4. The Matrix Effect and Spiked Recoveries

The matrix effect (ME) was evaluated using OPE-free plastic and textile samples (*n* = 10), which were extracted under the optimized conditions. Five nonspiked final extracts were directly run in the LC-MS/MS system, while the other five extracts were analyzed after being spiked with 10 ng of the native and isotopic standard. The standard in acetonitrile solution with the concentration of OPEs at 10 ng/mL was also analyzed simultaneously with the final extracts. The ME was calculated based on the peak areas of nonspiked and spiked extracts (*A*_nonspiked_ and *A*_spiked_) and the standard solution (*A*_standard_) using the equation: ME% = (*A*_spiked_ − A_nonspiked_)/A_standard_ *∗* 100. As shown in Figure S1, the MEs of OPEs were ranging from 62.4% to 117.0%, indicating that most OPEs display a little matrix suppression, which did not influence their detection, after the extraction and purification under the optimized conditions.

Spiking experiment was carried out by adding three different concentration levels (5, 10, and 50 ng/g) of the OPEs to the OPE-free plastic and textile samples, and the analysis was then run under the optimized conditions to verify the effectiveness and precision of the method. Each concentration level was tested in 3 replicates. Table 1 shows that the recoveries of the target substances ranged in 71.6%–114%, and the relative standard deviation was 0.8%–11.2%. Therefore, the developed method has good recovery and precision and is suitable for actual analysis.

### 3.5. Analysis of Actual Samples

The established method is applied to the analysis of target OPEs in commercially available fall protection equipment (safety helmets and ropes). The safety helmets were divided into shell plastics and internal materials before extraction and analysis. The results showed that all OPEs were detected in the fall protection equipment except that TMP was not detected in safety helmets (Table 2). The internal and external composition characteristics of OPEs in safety helmets were similar, both mainly containing EHDPP (26.84–95.29 ng/g) with other substances amounting to 5.136 ng/g or less. All OPEs were detected in the tested safety ropes with a high level of EHDPP (11.07–175.69 ng/g), and the concentration of other OPEs in safety ropes ranged in 0.05–22.75 ng/g. One sample of safety rope showed very high TPhP level (815.53 ng/g) and high levels of TEHP (58.83 ng/g) and TnBP (55.86 ng/g). Kajiwara et al. [9] found that TPhP was the dominant OPE among OPEs detected in computer-related accessories and curtain fabrics, mainly because of the wide use of TPhP in related fields. As a result, the detection of OPEs (with a focus on TPhP) in indoor dust is heavily associated with computer screens and televisions. Our results show another important source for the presence of TPhP in dust and for OPE release.

## 4. Conclusion

By optimizing the extraction conditions, separation conditions, and mass spectrometry detection parameters, an analytical method was developed for testing 14 kinds of OPEs in fall protection equipment. The developed method features accelerated solvent extraction of the analytes followed by purification with a solid-phase extraction column and finally analysis by HPLC-MS/MS. The method is rapid, sensitive, and reproducible and can readily detect OPEs in fall protection equipment (e.g., safety helmets and ropes) made of related materials.

## Figures and Tables

**Table 1:** The spike recoveries and LODs of OPEs.

Analyte	Spiked with 5 ng/g				Spiked with 10 ng/g				Spiked with 50 ng/g				LOD (ng/g)	
	Plastic material		Textile material		Plastic material		Textile material		Plastic material		Textile material		Plastic material	Textile material
	Recovery (%)	RSD (%)	Recovery (%)	RSD (%)	Recovery (%)	RSD (%)	Recovery (%)	RSD (%)	Recovery (%)	RSD (%)	Recovery (%)	RSD (%)		
TMP	77.6	2.1	82.5	2.8	65.8	2.9	88.2	1.8	69.5	1.8	82.3	2.1	1.25	0.63
TEP	68.1	3.9	67.3	3.5	72.9	3.5	72.5	2.9	70.1	0.8	70.6	3.5	0.26	0.13
TPrP	85.7	1.2	99.6	2.4	90.2	2.8	92.3	2.4	79.2	2.6	82.3	1.8	0.06	0.03
TnBP	72.4	1.9	80.7	3.5	75.8	2	82.4	0.9	86.7	1.8	102	0.9	0.06	0.03
TiBP	75.7	5.1	108.5	5.6	102	1.8	85.9	1.5	75.3	3.5	88.5	2.3	0.03	0.015
TEHP	69.5	4.4	76.9	4.2	84.3	2.4	100.2	2.1	75.7	5.7	89.1	1.9	0.15	0.08
TBEP	75.8	2.7	80.2	3	92.4	6.7	71.6	5.8	78.3	2.6	75.7	5.2	0.03	0.015
TCPP	105.4	3.5	98.5	2.8	91	1.9	83.7	1.6	104.5	1.8	87.6	1.2	0.39	0.19
TCEP	81.5	4.5	85.3	6.8	88.9	10.1	112.6	8.6	100.4	7.6	102.8	9.5	0.42	0.21
TDCPP	65.2	8.1	72.4	7.5	78.6	3.6	79.5	3.1	74.7	2.6	88.2	2.3	0.23	0.12
TPhP	73.7	11.2	91.3	8.2	108	1.6	98.5	2.3	78.5	7.4	92.3	6.8	0.23	0.12
TMPP	83.4	2.3	75.8	3.5	88.9	2.9	85.2	1.5	63.7	5.1	74.2	5.2	0.04	0.02
CDPP	71.6	5.4	100.5	4.2	114	2.1	103	1.8	78.7	2.3	80.8	2	1.33	0.67
EHDPP	93.2	4.8	88.3	5.8	87.8	7.8	77.5	4.6	85.9	5.8	101	3.5	0.05	0.026

**Table 2:** The concentrations of OPEs in safety helmets and ropes (unit: ng/g).

	Safety helmet 1#		Safety helmet 2#		Safety rope 1#	Safety rope 2#	Safety rope 3#	Safety rope 4#
	Outside part	Inside part	Outside part	Inside part				
TMP	<LOD	<LOD	<LOD	<LOD	0.637	1.602	<LOD	<LOD
TEP	<LOD	0.189	1.041	3.079	4.66	8.621	11.71	12.33
TPrP	0.158	<LOD	0.176	<LOD	0.05	0.357	0.2	0.212
TnBP	<LOD	0.536	0.139	1.914	5.782	55.86	6.022	5.788
TiBP	<LOD	<LOD	<LOD	0.149	1.545	14.82	1.027	0.414
TEHP	0.52	0.287	1.259	<LOD	0.475	58.83	0.409	3.686
TBEP	0.133	0.661	0.835	<LOD	0.145	0.775	0.114	0.166
TCPP	1.722	5.136	4.265	4.039	12.07	22.72	19.61	22.75
TCEP	<LOD	3.513	<LOD	3.465	5.804	18.76	12.07	21.33
TDCPP	<LOD	1.061	<LOD	0.796	1.516	8.505	5.363	3.216
TPhP	2.98	0.626	3.759	0.423	6.028	815.53	2.956	2.855
TMPP	0.132	0.071	0.219	0.045	0.505	10.02	1.222	4.675
CDPP	2.081	<LOD	1.424	<LOD	<LOD	2.08	1.222	1.867
EHDPP	51.71	26.84	95.29	32.66	53.54	11.07	28.39	175.69

## Data Availability

The data used to support the findings of this study are included within the article.

## Supplementary Materials

Supplementary Materials Figure S1: the matrix effect of OPEs (%). Table S1: the general information of target OPEs. Table S2: the related MS parameters of OPEs.

## References

[1] Wei G.-L., Li D.-Q., Zhuo M.-N. (2015). Organophosphorus flame retardants and plasticizers: sources, occurrence, toxicity and human exposure. *Environmental Pollution*.

[2] Greaves A. K., Letcher R. J. (2017). A review of organophosphate esters in the environment from biological effects to distribution and fate. *Bulletin of Environmental Contamination and Toxicology*.

[3] Zhang T., Bai X.-Y., Lu S.-Y. (2018). Urinary metabolites of organophosphate flame retardants in China: health risk from tris (2-chloroethyl) phosphate (TCEP) exposure. *Environment International*.

[4] Zhang Q., Ji C., Yin X. (2016). Thyroid hormone-disrupting activity and ecological risk assessment of phosphorus-containing flame retardants by in vitro, in vivo and in silico approaches. *Environmental Pollution*.

[5] Dishaw L. V., Powers C. M., Ryde I. T. (2011). Is the PentaBDE replacement, tris (1, 3-dichloro-2-propyl) phosphate (TDCPP), a developmental neurotoxicant? studies in PC12 cells. *Toxicology and Applied Pharmacology*.

[6] Zhang Q., Lu M., Dong X. (2014). Potential estrogenic effects of phosphorus-containing flame retardants. *Environmental Science & Technology*.

[7] Yuan S., Han Y., Ma M. (2019). Aryl-phosphorus-containing flame retardants induce oxidative stress, the p53-dependent DNA damage response and mitochondrial impairment in A549 cells. *Environmental Pollution*.

[8] Stapleton H. M., Klosterhaus S., Eagle S. (2009). Detection of organophosphate flame retardants in furniture foam and U.S. house dust. *Environmental Science & Technology*.

[9] Kajiwara N., Noma Y., Takigami H. (2011). Brominated and organophosphate flame retardants in selected consumer products on the Japanese market in 2008. *Journal of Hazardous Materials*.

[10] Wang Y., Hou M., Zhang Q. (2017). Organophosphorus flame retardants and plasticizers in building and decoration materials and their potential burdens in newly decorated houses in China. *Environmental Science & Technology*.

[11] Ionas A. C., Ballesteros Gómez A., Uchida N. (2015). Comprehensive characterisation of flame retardants in textile furnishings by ambient high resolution mass spectrometry, gas chromatography-mass spectrometry and environmental forensic microscopy. *Environmental Research*.

[12] GB/T2812-2006 (2006). *The standard for safety helmet*.

[13] Campone L., Piccinelli A. L., Östman C., Rastrelli L. (2010). Determination of organophosphorous flame retardants in fish tissues by matrix solid-phase dispersion and gas chromatography. *Analytical and Bioanalytical Chemistry*.

[14] Tsao Y.-C., Wang Y.-C., Wu S.-F., Ding W.-H. (2011). Microwave-assisted headspace solid-phase microextraction for the rapid determination of organophosphate esters in aqueous samples by gas chromatography-mass spectrometry. *Talanta*.

[15] Truong J. W., Diamond M. L., Helm P. A., Jantunen L. M. (2017). Isomers of tris (chloropropyl) phosphate (TCPP) in technical mixtures and environmental samples. *Analytical and Bioanalytical Chemistry*.

[16] Castro V., Montes R., Quintana J. B., Rodil R., Cela R. (2020). Determination of 18 organophosphorus flame retardants/plasticizers in mussel samples by matrix solid-phase dispersion combined to liquid chromatography-tandem mass spectrometry. *Talanta*.

[17] Du B., Zhang Y., Chen H., Shen M., Zhou W., Zeng L. (2019). Development and validation of a liquid chromatography-tandem mass spectrometry method for the simultaneous determination of 17 traditional and emerging aryl organophosphate esters in indoor dust. *Journal of Chromatography A*.

[18] Gao L., Shi Y., Li W., Ren W., Liu J., Cai Y. (2015). Determination of organophosphate esters in water samples by mixed-mode liquid chromatography and tandem mass spectrometry. *Journal of Separation Science*.

[19] Yang J., Zhao Y., Li M., Du M., Li X., Li Y. (2019). A review of a class of emerging contaminants: the classification, distribution, intensity of consumption, synthesis routes, environmental effects and expectation of pollution abatement to organophosphate flame retardants (OPFRs). *International Journal of Molecular Sciences*.

[20] Shi Y., Gao L., Li W., Wang Y., Liu J., Cai Y. (2016). Occurrence, distribution and seasonal variation of organophosphate flame retardants and plasticizers in urban surface water in Beijing, China. *Environmental Pollution*.

[21] Cao D., Guo J., Wang Y. (2017). Organophosphate esters in sediment of the Great Lakes. *Environmental Science & Technology*.

[22] Li W., Shi Y., Gao L., Wu C., Liu J., Cai Y. (2018). Occurrence, distribution and risk of organophosphate esters in urban road dust in Beijing, China. *Environmental Pollution*.

[23] Yang F., Ding J., Huang W., Xie W., Liu W. (2014). Particle size-specific distributions and preliminary exposure assessments of organophosphate flame retardants in office air particulate matter. *Environmental Science & Technology*.

[24] Li J., Zhao L., Letcher R. J. (2019). A review on organophosphate ester (OPE) flame retardants and plasticizers in foodstuffs: levels, distribution, human dietary exposure, and future directions. *Environment International*.

[25] García-López M., Rodríguez I., Cela R. (2009). Pressurized liquid extraction of organophosphate triesters from sediment samples using aqueous solutions. *Journal of Chromatography A*.

