# Supplementary material for: Determination of Organophosphorus Esters in Fall Protection Equipment by Accelerated Solvent Extraction and Solid-Phase Extraction Coupled with LC-MS/MS Detection
Source: J Anal Methods Chem. 2021 Jan 5;2021:8878247. doi: 10.1155/2021/8878247 (PMC7803397; doi:10.1155/2021/8878247)
Supplement: Supplementary Materials — Figure S1: the matrix effect of OPEs (%). Table S1: the general information of target OPEs. Table S2: the related MS parameters of OPEs. [file 8878247.f1.docx]

**Supporting Information**

**Determination of Organophosphorus Esters in Fall Protection Equipments by Accelerated Solvent Extraction and Solid Phase Extraction Coupled with LC-MS/MS Detection**

Li haihong^a,d^, Ye Mingli^b, c,*^, Wu Fangfang^a,d^, Zhao Xuyang^a, d^, Wang Lifeng^a, d^, Wei YiLi^a, d^, Xie Shengyi^a, d^, Hairong Cui^b,*^

^a^ Zhejiang Key Laboratory for Protection Technology of High-Rise Operation, Zhejiang Huadian Equipment Testing Institute Co. LTD, Hangzhou, Zhejiang, 310015, China;

^b^ School of life science of Wuchang university of Technology, Wuhan, Hubei, 430223, China;

^c^ College of Biological and Environmental Engineering, Zhejiang Shuren University, Hangzhou, 310015, China

^d^ SGCC-Testing Technology Lab of Electrical Equipment Safety Performance, Hangzhou, Zhejiang, 310015, China;

*Corresponding author:

Mingli Ye:

Tel: +86 (571)88297097; Fax: +86(571)888297098; Email: 2858749716@qq.com

Hairong Cui:

+86(27) 81652954; Fax: +86(27) 81652011; Email: 3224891342@qq.com

**Table S1 The general information of target OPEs**

| Abbreviation | Full name | Formulae | Molecular weight | CAS number |
| --- | --- | --- | --- | --- |
| TMP | Trimethyl phosphate | C3H9O4P | 140.08 | 512-56-1 |
| TEP | Triethyl phosphate | C6H15O4P | 182.16 | 78-40-0 |
| TPrP | Tripropyl phosphate | C9H21O4P | 224.23 | 513-08-06 |
| TnBP | Tri-n-butyl phosphate | C12H27O4P | 266.31 | 126-73-8 |
| TiBP | Tri-iso-butyl phosphate | C12H27O4P | 266.31 | 126-71-6 |
| TEHP | Tri(2-ethylhexyl) phosphate | C24H51O4P | 434.63 | 78-42-2 |
| TBEP | Tri(2-butoxyethyl) phosphate | C18H39O7P | 398.47 | 78-51-3 |
| TCPP | Tri(1-chloro-2-propyl) phosphate | C9H18Cl3O4P | 327.57 | 13674-84-5 |
| TCEP | Tri(2-chloroethyl) phosphate | C6H12Cl3O4P | 285.49 | 115-96-8 |
| TDCPP | Tri(1,3-dichloro-2-propyl) phosphate | C9H15Cl6O4P | 430.90 | 13674-87-8 |
| TPhP | Tri-phenyl phosphate | C18H15O4P | 326.28 | 115-86-6 |
| TMPP | Trimethylphenyl phosphate | C21H21O4P | 368.36 | 563-04-2 |
| CDPP | Cresyl diphenyl phosphate | C19H17O4P | 340.31 | 26444-49-5 |
| EHDPP | 2-Ethylhexyl di-phenyl phosphate | C20H27O4P | 362.41 | 1241-94-7 |

**Table S2 The related MS parameters of OPEs**

| Analyte | Quantitative transition | | Declustering potential (V) | Collision Energy (eV) | Collision Cell Exit  Potential (V) |
| --- | --- | --- | --- | --- | --- |
| TMP | 141.1 | 109.1* | 60 | 22 | 10 |
|  | 141.1 | 79.0 | 60 | 29 | 6 |
| TEP | 183.0 | 99.0* | 54 | 24 | 7 |
|  | 183.0 | 81.0 | 60 | 50 | 8 |
| TPrP | 225.4 | 99.0* | 60 | 22 | 7 |
|  | 225.4 | 141 | 60 | 24 | 10 |
| TnBP | 267.4 | 99.0* | 60 | 20 | 10 |
|  | 267.4 | 155 | 60 | 12 | 10 |
| TiBP | 267.4 | 99.0* | 60 | 20 | 10 |
|  | 267.4 | 155 | 60 | 12 | 10 |
| TEHP | 435.3 | 99.0* | 115 | 32 | 6 |
|  | 435.3 | 113.1 | 120 | 16 | 8 |
| TBEP | 399.3 | 299.3* | 95 | 19 | 10 |
|  | 399.3 | 199.0 | 95 | 21 | 10 |
| TCPP | 327.0 | 99.0* | 70 | 30 | 10 |
|  | 329.1 | 99.0 | 70 | 28 | 10 |
| TCEP | 285.0 | 99.0* | 80 | 42 | 10 |
|  | 287.0 | 99.2 | 75 | 30 | 10 |
| TDCPP | 431.1 | 98.9* | 85 | 35 | 9 |
|  | 431.1 | 208.9 | 84 | 20 | 8 |
| TPhP | 327.1 | 215.0* | 130 | 42 | 11 |
|  | 327.1 | 153.1 | 130 | 65 | 7 |
| TMPP | 369.2 | 166.1* | 147 | 37 | 11 |
|  | 369.2 | 90.9 | 147 | 61 | 8 |
| CDPP | 341.1 | 152.1* | 135 | 40 | 10 |
|  | 341.1 | 165.1 | 135 | 40 | 10 |
| EHDPP | 363.2 | 251.0* | 70 | 71 | 7 |
|  | 363.2 | 152.9 | 72 | 12 | 9 |
| d9-TMP | 150.1 | 115.1 | 90 | 24 | 10 |
| d15-TEP | 198.1 | 101.9 | 65 | 27 | 8 |
| d21-TPRP | 246.4 | 102 | 120 | 25 | 9 |
| d12-TCEP | 299.1 | 102 | 75 | 30 | 6 |
| d27-TnBP | 294.4 | 102 | 140 | 25 | 10 |
| d15-TPhP | 342.2 | 161.3 | 135 | 75 | 9 |
| d18-TCPP | 345.1 | 102 | 75 | 30 | 8 |

*The transition for quantification


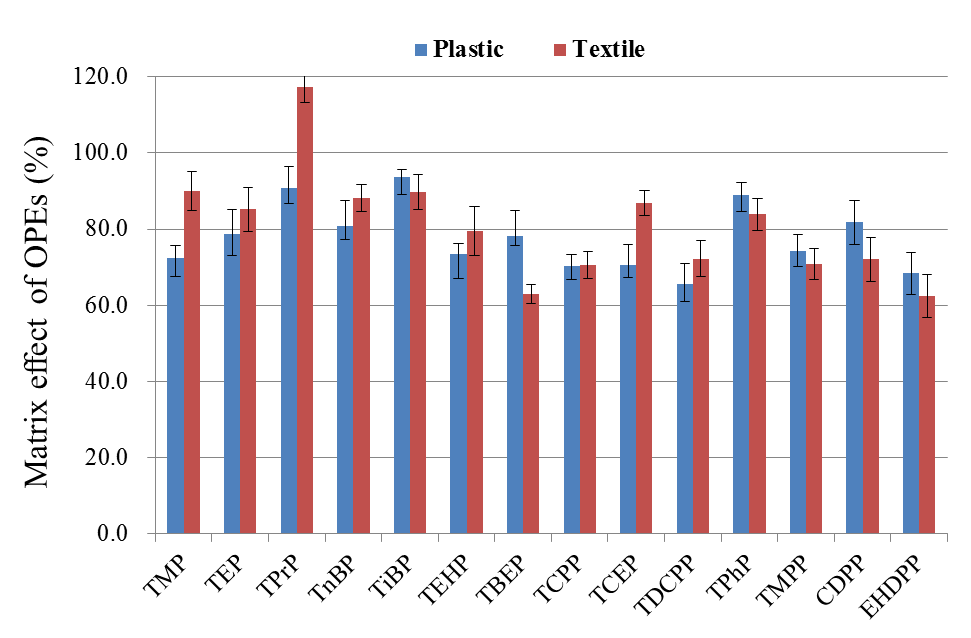


**Figure S1 The matrix effect of OPEs (%)**
